# Managing expansions in medical students’ clinical placements caused by curricular transformation: perspectives from four medical schools by

**DOI:** 10.1080/10872981.2020.1857322

**Published:** 2020-12-17

**Authors:** Jeff A. Kraakevik, Gary L. Beck Dallaghan, Julie S. Byerley, Seetha U. Monrad, John A. Davis, Maya M. Hammoud, Cyril M. Grum, Patricia Carney

**Affiliations:** a School of Medicine, Oregon Health & Science University, Portland, OR, USA; b School of Medicine, University of North Carolina, Chapel Hill, NC, USA; d University of Michigan, Ann Arbor, MI, USA; e School of Medicine, University of California, San Francisco, CA, USA; f University of Michigan Medical School, Ann Arbor, MI, USA

**Keywords:** Curriculum transformation, clerkship

## Abstract

Many challenges could occur that result in the need to handle an increase in the number of medical student clinical placements, such as curricular transformations or viral pandemics, such as COVID 19. Here, we describe four different institutions’ approaches to addressing the impact of curricular transformation on clerkships using an implementation science lens. Specifically, we explore four different approaches to managing the ‘bulge’ as classes overlap in clerkships Curriculum leaders at four medical schools report on managing the bulge of core clinical placements resulting from reducing the duration of the foundational sciences curriculum and calendar shifts for the respective clerkship curriculum. These changes, which occurred between 2014 and 2018, led to more students being enrolled in core clinical rotations at the same time than occurred previously. Schools provided respective metrics used to evaluate the effectiveness of their bulge management technique. These data typically included number of students affected in each phase of their curricular transformation, performance on standardized examinations, and student and faculty feedback. Not all data were available from all schools, as some schools are still working through their ‘bulge’ or are affected by COVID-19. There is much to be learned about managing curricular transformations. Working on such endeavors in a learning collaborative such as the AMA Accelerating Change in Medical Education Initiative provided support and insights about how to survive, thrive and identifying lessons learned during curricular transformation.

## Introduction

Innovative educational transformations are occurring across physician training, including undergraduate medical education (UME) [1–3] and graduate medical education (GME) [4–7]. Some curricular revisions occurring in UME involve decreasing basic science education delivered before core clerkships, which can result in an influx of medical students entering clinical rotations earlier than occurred in the prior curriculum, creating a ‘bulge’ in student clinical placements. Such transformations can stress faculty, staff, residents, and medical students, especially when two different curricula are running simultaneously. In the age of COVID-19, the pressure on securing clinical placements or doing them virtually is intensifying in terms of complexity (PC1- 8, PC2 − 9).

The American Medical Association’s (AMA) Accelerating Change in Medical Education Consortium [8] is a learning collaborative formed in September 2013, initially including 11 medical schools and expanding to 32 schools in January 2016. This ongoing collaborative was designed to share ideas across North American medical schools implementing innovations. The innovation projects included, but were not limited to, developing flexible, competency-based educational pathways; teaching and evaluating new content in health systems science; working with healthcare delivery systems in novel ways to educate students; creating individualized coaching models to guide learners through their educational processes; and leveraging technology to support learning and assessment [9]. The four schools represented in this paper united around the fact that we were assessing the impact curricular changes had in some detail.

Though theoretical work in implementation science around curricular transformations exists [10–12], specific applications examining how challenges are solved while rigorously assessing outcomes are rare. Given the complexities and cultures unique to different educational institutions, it is unlikely that simple answers to challenging transformation problems exist; thus, one size will not fit all. With this in mind, we applied a determinant framework from implementation science [13] and analyzed institutional and individual facilitators and/or barriers relevant to anticipated student outcomes associated with managing curricular transformation.

The purpose of this paper is to describe, through the lens of implementation science, how four medical schools in the AMA Consortium (Oregon Health & Science University [OHSU], University of North Carolina at Chapel Hill [UNC SOM], University of Michigan [UMMS], and the University of California, San Francisco [UCSF]) managed the bulge in student placements resulting from curricular innovations and calendar changes. Participating schools that present student data underwent IRB reviews and received approvals and/or exemptions (OHSU: IRB # 10,873- Approval; UMMS: HUM00130655-Exempt/Approved; UNC SOM: IRB # 18–2165-Exempt).

## Theoretical framework

Determinant frameworks in implementation science [13] have been used to describe facilitators and/or barriers that influence innovation implementation and outcomes. The frameworks recognize intersecting relationships and determinants or factors within systems that make implementing innovations a multidimensional phenomenon. This aids in identifying strategies likely to foster enhanced outcomes. Determinants are classified using the Consolidated Framework for Implementation Research (CFIR) [14]. The CFIR is comprised of five domains, including the intervention, setting, individuals, and process of enacting the implementation. Implementations can be characterized as having core or essential components as well as an adaptable periphery (elements that are adaptable to the intervention being implemented) [14]. With this in mind, we defined the following domains to include structural characteristics; logistical characteristics; and networks and communication, all of which were adaptable. For the purposes of this paper, we briefly describe both the curricular changes undertaken by the four universities and key determinants and outcomes resulting from implementation.

## Structural characteristics (adaptable component of implementation)

Each university managed curricular restructuring differently (Table 1). All four did compress the pre-clinical curriculum but used different approaches. For example, OHSU removed their summer break, so the curricular hours did not change but students completed it earlier in their program. Each school did handle the influx of additional students in their respective clinical structures in adaptable ways.

**Table 1.** Characteristics of medical school curricular changes by institution

	Oregon Health & Science University	University of North Carolina	University of Michigan	University of California, San Francisco
# Students in entering class Last year of prior curriculum First year of new curriculum	147 144	180 180	171 170	150 (165) 150 (165)
Academic Year New Curriculum Initiated	2014	2014	2014	2016
Academic Year New Curriculum Fully Implemented	2018	2018	2017	2020
Number of Months to Complete Pre-Clerkship Education Phase	18	18	12	17
Timing of USMLE Step I in Old Curriculum	Pre-clerkship	Pre-clerkship	Pre-clerkship	Pre-clerkship
Timing of USMLE Step I in New Curriculum	Pre-clerkship	Pre-clerkship	Post-clerkship	Post-clerkship
Calendar month that new class enters clinical phase	March	March	October	January
Bulge Approach	Shortened core clerkship year by offering students in Neurology Clerkship the option to opt out by: 1) passing the NBME shelf examination in clinical neurology, and 2) completing a faculty-observed history and complete neurological examination.	Combined and shortened clerkships for last year of ‘old’ curriculum to total 36 instead of 48 weeks. Added 12 weeks required elective time to compensate.	Shortened core clerkship year by 25% (from 48 months to 36 months) for 3 consecutive cohorts, while simultaneously incrementally shortening preclerkship period (from 19 months to 16 months and then to 12 months)	Clerkship year for old curriculum extended by 4 months to allow for more even distribution. Old curriculum students were permitted to take 4^th^ year rotations during this time. Some new sites were added to key clerkships.
Number of months to complete required clerkships	17	9	9	12
Number of months planned for bulge resolution	2	4	27	6
Types of assessments/comparisons conducted	Students age, gender USMLE Step I raw score and pass rates NBME Internal Medicine shelf exam scores NBME neurology shelf exam scores Course materials accessed Students’ grades Students career choice for residency training End-of-course evaluation and Focus group with students facilitated by a UME curriculum committee liaison	Medical student feedback Medical school faculty feedback Staff feedback Expense Student shelf exam USMLE Step II performance Student career choice	Performance on clerkship exams Performance on end of year comprehensive clinical assessments Student evaluations of clerkship quality Student indices of well-being Step 1 performance	Comparisons planned for pre-transition, transition, and new curriculum students Clerkship-based assessment comparisons Clinical evals Shelf exams Standardized exams NBME CBSA Step 1 Step 2 (CS, CK) Clerkship-based and overall curricular student feedback End-of-clerkship surveys Quarterly focus groups End of clerkship phase survey

**Figure 1.**
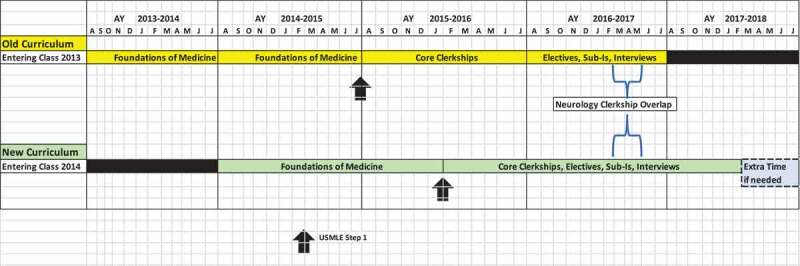
Oregon Health & Science University’s curricular transformation

**OHSU** underwent curriculum transformation to ‘YourMD’ in 2014 with the first class graduating in 2018. It was designed to provide flexibility for elective time, enrichment opportunities, tailored coaching, and was time-varying competency-based. The schedule for the transformation was dictated, in part, by receipt of the AMA grant [8] where the school committed to a rapid transformation to an entirely new curriculum in the first year of the grant. As part of this transformation, the first 2 years in the prior curriculum were reduced to 18 months (Figure 1), which resulted in doubling of the number of medical students per clinical rotation between March and May of 2017, when the overlap of the new and the old curriculum involved clinical rotations for twice the number of learners. Thus, the most significant bulge for OHSU’s transition was limited to 12 weeks.

**Figure 2.**
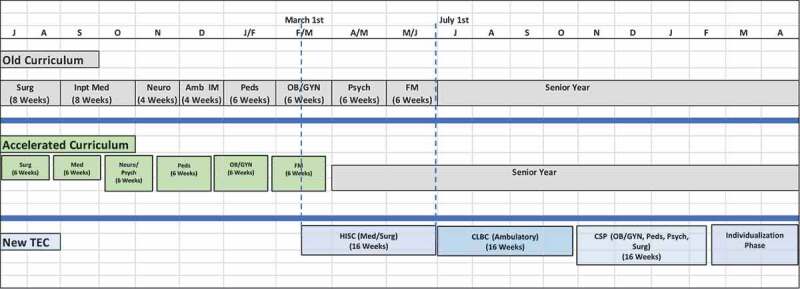
University of North Carolina’s Curricular Transformation – years 3 and 4 only

**UNC’s SOM** Translational Education at Carolina (TEC) curriculum was developed and implemented in Fall 2014. The Foundation Phase of TEC compressed the science classroom course work into three semesters, wrapping up in December of their second year. Students were provided unstructured time in January and February in Year 2 to prepare for USMLE Step 1, with clerkships beginning on March 1^st^ (Figure 2), with the clerkship being a 48-week experience. Due to implementing TEC in 2016, a 3-month bulge with 180 + 180 students in clerkships occurred. Complicating the situation was UNC SOM’s four different clinical curricula based at different campuses, each with varying lengths of longitudinal exposure to core clinical disciplines. This necessitated planning for overlap strategies in multiple sites. Three regional campuses depend on volunteer, community preceptors. Therefore, the bulge needed to be managed carefully to avoid losing training sites.

**Figure 3.**
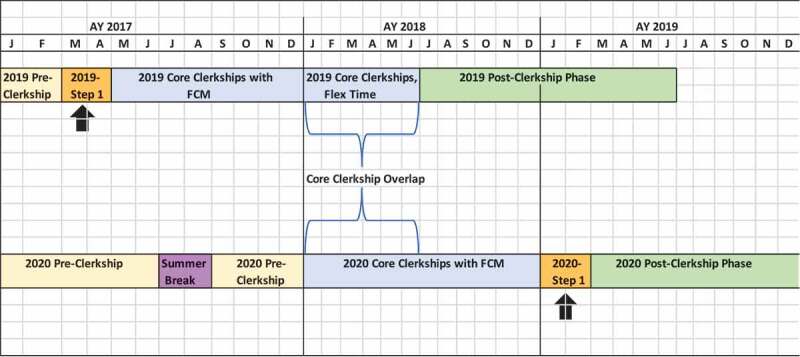
UCSF curricular transformation

Prior to 2016, the **UCSF** pre-clerkship phase ended in March of the second academic year, followed by USMLE Step 1, with the clerkship phase beginning in April of the second year. The UCSF Bridges Curriculum began in August 2016 and included an integration of foundational science instruction over the pre-clerkship and clerkship phases (Figure 3). The new pre-clerkship phase ended in December of Year 2, and UCSF moved Step 1 to after the clerkship phase. This permitted the start of the clerkship phase in January of the second academic year. As a result, during the transition from the old curriculum to the new, a bulge of two cohorts in the clerkships occurred for approximately 6 months (January – April).

**UMMS’s** curricular transformation began in 2014 and involved shortening the pre-clinical curriculum from 19 months to 12 months. If the transition occurred between 1 year and the next, such as is described for the preceding schools, there would have been a 7 month bulge of two cohorts in the clerkships.

## Logistical characteristics (adaptable component of implementation)

The capacity of patient care settings was taken into consideration when curricular decisions were made. Identifying approaches for dealing with the bulge required demonstrating advantages of the proposed changes to stakeholders [14]. A key approach involved innovative scheduling. Each university undertook different adaptable scheduling changes, with one school avoiding the bulge altogether.

**Figure 4.**
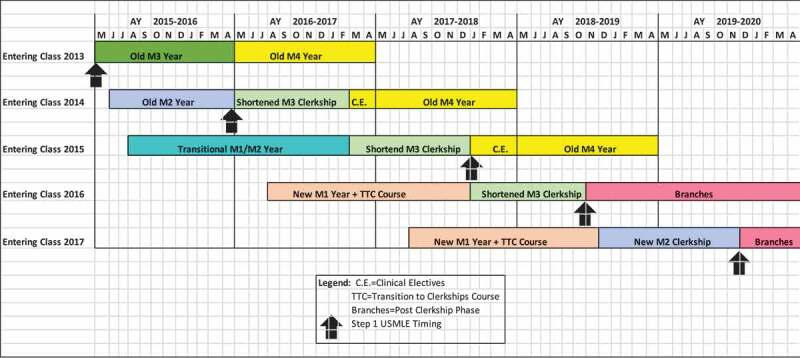
University of Michigan strategy for managing the bulge in clinical placements

**UMMS** avoided the bulge completely because a 7-month overlap of 2 cohorts of clerkship students (n = ~340) would have significantly compromised educational experiences for students and been exceedingly stressful for faculty, staff and institutional leaders. Thus, all clerkships were shortened by 25% for three consecutive years, reducing the clerkship period from 48 weeks to 36 weeks, while maintaining approximately 85% of didactics and specific clinical experiences. This resulted in no overlap in clerkship cohorts for the 27 months that the transition to the new curricular model took to complete (Figure 4).

Other approaches to curricular transformation involved either shortening or eliminating required experiences. For example, the **OHSU’s** neurology clerkship transitioned from a fourth-year required clerkship to a third-year required clerkship, which further complicated the bulge. In addition to identifying supplementary clinical sites to place students, a novel solution proposed by the neurology clerkship leadership was to offer 4^th^ year medical students in the prior curriculum the option of opting-out of the required neurology clerkship by: 1) passing the NBME subject examination in clinical neurology, and 2) completing a faculty-observed history and complete neurological examination. The goal was to reduce learner load by half of the graduating class while allowing for assessment of competency in clinical neurology. This proposal was approved by the OHSU UME curriculum committee, and a paper that fully describes the comprehensive assessment of this innovative approach is published elsewhere [15].

For the **UNC SOM** Accelerated Class, clerkships were shorted from 48 to 36 weeks by combining neurology and psychiatry into one 6-week experience, combining ambulatory internal medicine and family medicine into one 6-week experience, and shortening internal medicine and surgery from 8 to 6 weeks each. On the two campuses that were teaching in a longitudinal integrated clerkship model (Asheville and Charlotte), the time in the clerkships was shortened from 48 weeks to 36 weeks during the transition year. This resulted in only 4 weeks of overlapping cohorts in the core clerkships at the same time, which was considered an acceptable duration for clerkship directors and didactic leaders, and for clinical capacity to be tight as more students than typical were placed in various clinical settings. In exchange for 12 weeks shorter of clerkships, elective requirements were increased in fourth year by three 4-week block requirements. Thus, the total clinical curriculum was the same duration but the core clerkship time was shortened.

Because of the anticipated challenge of accommodating two classes of learners together for the several months of overlap at **UCSF**, several changes were made for the last year of the previous curriculum’s clerkship phase. These included two structural changes for the Bridges Clerkship year, uncoupling of the neurology and psychiatry clerkships and changing the longitudinal nature of the family and community medicine (FCM) clerkship, all of which were piloted, adding to the flexibility of the schedules in the last class of the previous curriculum. Previously, the neurology and psychiatry clerkships were coupled into one 8-week block. The Bridges Curriculum uncoupled them and instead coupled them with elective time, increasing the logistic flexibility of student schedules. Also, in the previous curriculum, the FCM clerkship was an 8-week block. This was integrated into the curriculum to be longitudinal starting with the last class of the previous clerkship.

Due to limitations in the number of FCM clinical sites, there could be no overlap in the classes on that clerkship, so the FCM clerkship was shortened to end in December by accelerating the schedule of clinical days (once a week instead of once every other week). Additionally, the clerkship year of the previous curriculum was extended by 2 months to allow additional flexibility, including a mix of fourth year electives and core clerkships (Figure 3). While these interventions minimized the number of additional learners each individual clerkship had to accommodate, it still meant that there were clerkship students of two different curricula rotating on the wards for approximately 6 months (January through June). This minimized the overlap of students doing core clerkships at the same time.

Simultaneously, rather than ending the clerkship phase of the last year of the previous curriculum two months early (due to the longitudinal integration of FCM), the end of the phase was kept the same, increasing the overall overlap period, and decreasing the number of students experiencing a schedule conflict (essentially averaging the ‘bulge’ over two additional months).

## Networking & communications (adaptable component of implementation)

Effective communication strategies are essential for implementing change [14]. Structural organization and meticulously outlined plans are dependent on clearly communicated goals. Careful consideration was made to prepare faculty and students about these curricular changes, though each school adapted a strategy that would work best for them.

Special attention was paid to the **UNC SOM** class that was to be the last cohort of the traditional curriculum. The administrative leaders and curriculum committee were concerned the students might feel neglected and vulnerable; thus, they labeled them ‘The Accelerated Class’, explaining to them that they were accelerating into some of the benefits of the upcoming TEC curriculum. This specific labeling focused attention on this class’ needs. Additionally, UNC SOM clerkship directors carefully planned faculty interactions during this intense month to ensure both the Accelerated Class and the first class of TEC were well attended. Extensive faculty development was provided during that time and frequent communications at the School, Department, and Clerkship level ensured familiarity with the differing needs of the student groups. To ensure clinical capacity, additional clinical sites were cultivated where possible. New electives were developed and capacity within electives and selectives was enhanced through active work with departmentally based faculty. This was helpful in preparing for the TEC curriculum where additional clinical rotations were needed for the longer individualization phase.

Transforming experiences into a longitudinal experience at **UCSF** required communication around the overlap period. As students from two different curricula were on clinical clerkships simultaneously, a specific strategy for distinguishing level of learner was developed, which included color distinctions on ID badges so that educators could quickly determine the curriculum of a given medical student. Students in both cohorts were counseled in advance about the overlap, and how to communicate their curriculum preparation to educators who might not have been familiar with it. Just-in-time online videos were available to faculty who were serving as educators on the clerkships, and emails and other communications materials (posters, flyers) were disseminated to help educators of all levels with both the new curriculum and how to work with both cohorts of students.

Students in the last year of the previous curriculum had additional elective time in their clerkship year that had not previously been there. The students desired taking traditionally fourth-year electives in that timeframe. This required communication with those departments about their stated requirements, and careful counseling of students about what electives would be appropriate, given the preparation of each individual student. Lastly, opportunities were crafted to allow previous curriculum students to communicate to the first Bridges Curriculum cohort, allowing for sharing of experiences, piloting some of the curricular changes, and adding transparency around improvements to those curricular elements made due to the feedback of students in the previous curriculum.

A communications team assisted **OHSU** in assuring faculty and staff were aware of updates throughout the curriculum transformation process. This communication team strategized discussions of the transition process to those within the academic community as well as to volunteer faculty throughout the state. The specific method of how bulge students were handled was left to the discretion of the individual clerkship director. Many of these directors kept students from the two overlapping classes assigned to separate sites with communication to the sites left to the clerkship coordinator for the 12-week overlap. The neurology clerkship utilized an advisory group of key faculty members to help with communicating the new curricular elements to the faculty in their subspecialty groups. **UMMS** had a communication strategy similar to OHSU’s.

## Findings

By explaining structural characteristics, logistical characteristics, and networks and communication for each institution, we have laid the foundation to explore the domains of CFIR related to these curricular innovations. As noted previously, CFIR involves five domains: intervention, inner and outer settings, individuals involved, and intervention process [14]. The following discussion goes into further detail for each of these domains.

The intervention across all four institutions involved shortening the pre-clinical curriculum. The AMA Accelerating Change in Medical Education support allowed these schools to undertake the curricular innovations, but it also presented a challenge to address the bulge of students in clinical settings. Because of this influx of students on clinical rotations, the actual intervention process was managed in unique and adaptable ways by each institution. For example, UMMS avoided an influx altogether by shortening all of their clerkships over a 3-year period of time while OHSU and UNC opted to shorten required experiences.

The outer setting has been explained as the economic, political, and social context within which the institution resides while the inner setting involves the structural, political and cultural contexts through which the implementation proceeds [14]. Often times, there is not a hard line between the inner and outer settings. For all four medical schools, the inner setting involved carefully addressing structural and logistical characteristics to ensure support from leadership and clinical educators impacted by the adjustments to their clerkships. In addition, structural and logistical characteristics needed to be addressed with clinical partners, who may be part of the inner and outer setting to successfully manage the modified clinical experiences.

Networks and communication were employed to ensure individuals involved with the implementation of the new curriculum had a voice and could offer inputs. This was particularly important for clinical educators and administrative staff to be engaged due to changing schedules and accelerated rotations. More importantly, medical students are the individuals involved that were of utmost concern. The curricular changes could not undermine their experiences. Therefore, each school has used different adaptive metrics to evaluate the impact of the curriculum on their students, which are detailed below.

The first shortened clerkship cohort (2014 matriculants) at **UMMS** experienced a 25% reduction in clerkship duration alone without any other major curricular changes. Student performance on NBME clerkship subject exams remained stable, with only the Pediatrics clerkship experiencing a statistically significant, but slight, decline [16]. Students handled the rapid paced shortened clerkships quite well. Their perceptions of clerkship quality, stress and well-being, and performance on an end-of-clerkship year multi-station clinical competency assessment stayed the same and/or improved slightly [16]. Anecdotally, the change was more burdensome for administrative staff; especially tracking students with a continuously changing academic calendar.

The second cohort (2015 matriculants) took Step 1 after clerkships, in addition to experiencing a shortened clerkship duration and a slightly shortened pre-clerkship curriculum. UMMS findings showed a decrease in shelf scores in Internal Medicine, Family Medicine, Pediatrics and Surgery compared with the preceding cohort that took Step 1 prior to clerkships. However, when comparing performance between comparable periods over the course of the clerkship years, the difference resolved for all clerkships except Pediatrics [17]. Additionally, this cohort experienced an increase in Step 1 scores compared with previous cohorts who took Step 1 prior to the clerkship, in line with other reports on the impact of moving USMLE Step 1 to after the clerkships [18]. The third cohort (2016 matriculants) has completed the end of their shortened clerkship phase and analyses are underway. They experienced a one-year preclinical curriculum, in addition to not taking Step 1 prior to clerkships.

**OHSU**’s decision to allow students to opt out of the Neurology clerkship was also a unique adaptive way of addressing the bulge. Of 133 fourth-year students in academic year 2016–2017, 57 (42.9%) chose to complete the required neurology clerkship and 77 (57.9%) chose to opt out [15]. Other detailed findings from this strategy are reported elsewhere [15], including that choosing to opt out appeared to be associated with residency training discipline where those choosing medical residencies more likely to take the clerkship compared to those undertaking surgical residencies [15]. Students who opted out either took electives or undertook residency interview visits. Comments from the focus group of OHSU opt-out student learners indicated: 1) they liked flexibility it allowed in schedule; 2) they liked the 1:1 neuro-exam instruction/feedback/evaluation session as they were able to learn how to improve exam skills; 3) while those who opted-out were glad they chose this option, they did feel their overall competency for caring for neurological patients was not equivalent to their colleagues who took the required clerkship. Based on this, educators at OHSU concluded that opting out of the clerkship was a feasible option for a fourth-year to third year curricular transition as it reduced learner load. Other neurology clerkships are likely going to go through similar transition as evidenced by changes seen in AAN Neurology Clerkship director survey, indicating an increased number of third-year required clerkships from 45% to 56% over 7 years [19].

**UCSF** is currently in the midst of its bulge period. Learner satisfaction data from both classes involved in the bulge will be compared to standard data gathered on classes before and after the bulge period. Learning outcomes (clinical performance and measures of medical knowledge) for learners involved in the bulge as compared with others who had been through the same curriculum (whether our new Bridges curriculum or the Traditional curriculum) without the bulge will also be compared.

The **UNC SOM** considered traditional outcome measures to monitor student progress during this transition time. Given the accelerated timeframe of the clerkships, we tracked student performance on their NBME Subject Examinations by specialty. In reviewing scores over a 3-year period (pre-transformation, accelerated year, TEC curriculum), Family Medicine saw an improvement in scores across all 3 years. There was no difference in Neurology clerkship scores. For Medicine, OB/GYN, Pediatrics, Psychiatry, and Surgery, scores dipped during the accelerated year, but have since rebounded and are comparable to the pre-transformation scores. Additionally, USMLE Step 2 scores on average did not see a significant change from the accelerated year and have gone up in the TEC curriculum.

## Conclusions

Implementation science frameworks have largely been addressed in theory rather than application [11,13,14]. We have shown how such frameworks can be applied at four universities as part of their work analyses, specifically for adaptive processes involved in implementation, the context within which it occurred, and the setting.

Curricular transformation forces institutions to deal with an overload of clinical students and strategies are worth considering for long-term management of placing large number of students. We have presented four different strategies to deal with student load during clinical rotations depending on local circumstances employing concepts from implementation science. Although only four schools are represented here, the diverse methods initiated to manage the influx of additional students for clinical placements are unique. Yet, based on different measures of student performance and faculty and student feedback, these approaches were all successful in their own right, primarily because they were adaptive to the unique features of each institution. Given, we are in the midst of the COVID-19 pandemic, many schools are having to handle bulges in students’ clinical placements [20,21] and it is likely that each will need to adapt its strategies in ways that will work best for their students, faculty and staff. Future studies are needed to determine how these students later perform during residency and into independent clinical practice. As medical schools consider undertaking restructuring their curriculum, use of implementation science frameworks can optimize the planning and execution of robust changes to the clinical enterprise and offer students a sound educational experience.

## Practice points

- Managing student placements during curricular transformation requires several strategies
- Planning and communicating carefully are required
- Evaluating strategies used is important for students, faculty and institutional leaders
